# Macrophage Function and the Role of GSK3

**DOI:** 10.3390/ijms22042206

**Published:** 2021-02-23

**Authors:** Sarvatit Patel, Geoff H. Werstuck

**Affiliations:** 1 Thrombosis and Atherosclerosis Research Institute, 237 Barton Street E, Hamilton, ON L9L 2X2, Canada; sarvatit.patel@taari.ca; 2 Department of Chemistry and Chemical Biology, McMaster University, 1280 Main St W, Hamilton, ON L8S 4L8, Canada; 3 Department of Medicine, McMaster University, 1280 Main St W, Hamilton, ON L8S 4L8, Canada

**Keywords:** macrophage function, glycogen synthase kinase (GSK)-3, molecular mechanisms, inflammatory response, atherosclerosis

## Abstract

Macrophages are present in nearly all vertebrate tissues, where they respond to a complex variety of regulatory signals to coordinate immune functions involved in tissue development, metabolism, homeostasis, and repair. Glycogen synthase kinase 3 (GSK3) is a ubiquitously expressed protein kinase that plays important roles in multiple pathways involved in cell metabolism. Dysregulation of GSK3 has been implicated in several prevalent metabolic disorders, and recent findings have highlighted the importance of GSK3 activity in the regulation of macrophages, especially with respect to the initiation of specific pathologies. This makes GSK3 a potential therapeutic target for the development of novel drugs to modulate immunometabolic responses. Here, we summarize recent findings that have contributed to our understanding of how GSK3 regulates macrophage function, and we discuss the role of GSK3 in the development of metabolic disorders and diseases.

## 1. Macrophages

**Figure 1:**
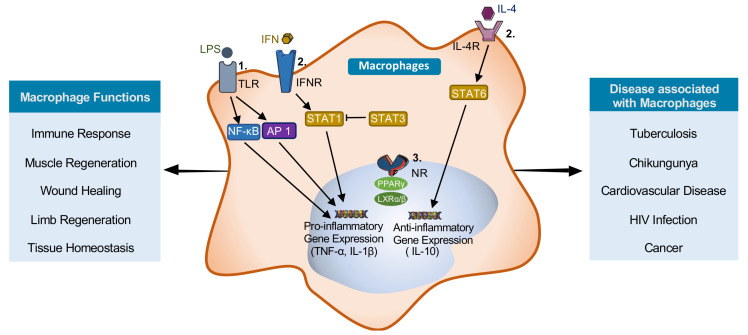
Macrophage: polarization, function, and associated diseases. Three main families of receptors regulate macrophage polarization and function. These are (1) the toll-like receptors (TLRs) that signal through nuclear factor kappa-light-chain-enhancer of activated B cells (NFκB) and AP-1, (2) the interferon receptor (IFNR) and interleukin (IL)-4 receptors that signal through signal transducer and activator of transcriptions (STATs), and (3) the nuclear receptors. Macrophage stimulated with lipopolysaccharide (LPS) and/or interferon-gamma (IFNγ) polarize to pro-inflammatory (M1) macrophages. Stimulation with IL4 induce polarization to anti-inflammatory (M2) macrophages. Regulation is important for proper physiological responses; however, dysregulation can contribute to the pathogenesis of diseases.

Macrophages are the first line of defense in the innate immune system. While most macrophages differentiate from circulating monocytes, a distinct embryonically derived population of resident macrophages exists in many tissues, including the heart, lung, and liver [1]. Macrophages play versatile phagocytic, endocytic, and secretory roles as a central part of the maintenance of tissue homeostasis [2], wound healing [3], muscle regeneration [4], and limb regeneration [5] (Figure 1). In response to chemotactic signals, they migrate toward sites of inflammation, where they ingest and degrade cell debris and orchestrate inflammatory responses. Macrophage function is dysregulated in several diseases, including tuberculosis [6], chikungunya [7], cardiovascular disease [8], HIV infection [9], cancer [10], and obesity [11].

Tissue-specific macrophage activity is regulated by microenvironmental stimuli that direct distinct transcriptional programming to modulate macrophage function [12]. There are three broad groups of receptors that are particularly relevant for macrophage activation (Figure 1): (i) receptors coupled to the nuclear factor kappa-light-chain-enhancer of activated B cells (NF-κB) and activator protein 1 (AP-1) family of transcription factors, which regulate most inflammatory genes; (ii) receptors coupled to signal transducer and activator of transcription (STAT) family transcription factors; and (iii) nuclear receptors (NR) regulating transcriptional activity [12]. Signaling through these pathways determines macrophage polarization and phenotype.

Macrophages can be polarized into several different subtypes that have distinct characteristics/functions. The extreme phenotypes are pro-inflammatory (M1) macrophages and anti-inflammatory (M2) macrophages [13]. Pro-inflammatory macrophages can be induced by exposure to interferon-gamma (IFNγ) and/or lipopolysaccharide (LPS). These macrophages play an important role early in a crisis by mobilizing a response to localized injury. Pro-inflammatory macrophages express transcription factors such as NF-κB, AP-1, STAT1, and interferon regulatory factor (IRF)-5, which leads to increase secretion of inflammatory cytokines including tumor necrosis factor (TNF)-α, interleukin (IL)-1β, IL-6, and IL-12 [13]. This amplifies the inflammatory response and directs host-defenses against invading pathogens. Anti-inflammatory, or alternatively activated, macrophages can be induced by exposure to IL-4 or IL-13. Alternatively, activated macrophages produce anti-inflammatory cytokines, including IL-10 and transforming growth factor (TGF)-β [13]. These anti-inflammatory macrophages are important for resolving inflammation, initiation of tissue repair, and return to tissue homeostasis [14]. There are two main groups of anti-inflammatory macrophages, regulatory macrophages, and wound-healing macrophages [15]. Regulatory macrophages facilitate the resolution of inflammation through the secretion of the immunosuppressive cytokine IL-10 [15]. The wound-healing macrophages produce IL-4 and exhibit enhanced arginase activity to produce polyamines and collagen in order to facilitate the redevelopment of the damaged tissue [15]. Several intermediate macrophage subtypes, including M_ox_, M_hem_, and M_4_, have been identified that each expresses a unique combination of markers [16]. The role and importance of these macrophage subtypes are less well understood [16]. Exogenous control over macrophage polarization may facilitate modulation of the inflammatory response and more efficient wound healing and tissue regeneration.

## 2. Glycogen Synthase Kinase 3

GSK3 is a serine/threonine kinase that plays a central role in several pathways that regulate cell metabolism, proliferation, and viability [17]. There are two ubiquitously expressed forms of GSK3 in mammals (GSK3α (51 kDa) and GSK3β (47 kDa)), as well as the GSK3β splice variant, GSK3β2, which is expressed primarily in the central nervous system [18]. Isoforms GSK3α and GSK3β are 98% homologous within the kinase domain and appear to possess both overlapping and unique functions [19]. Over one hundred putative substrates for GSK3α/β have been identified [20]; however, the physiological relevance of most of these is not known.

**Figure 2:**
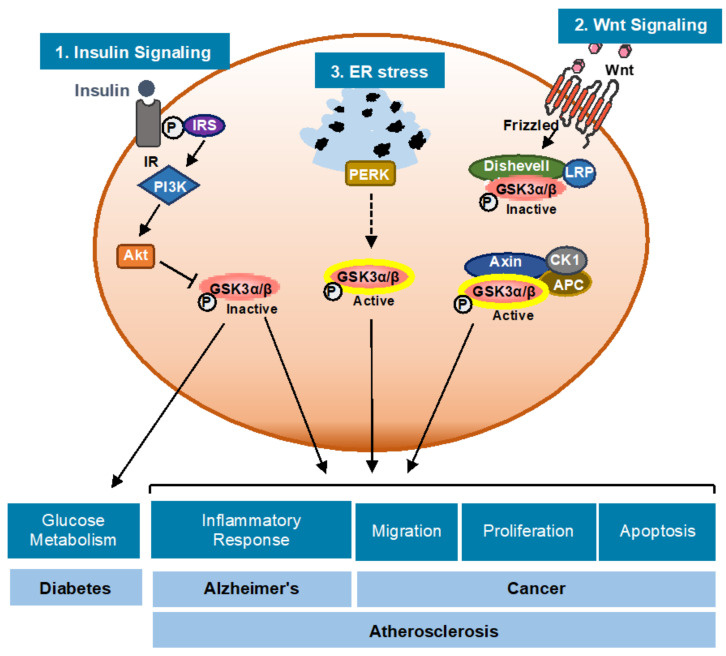
GSK3α/β: regulation, cellular functions and diseases. Three cellular signaling pathways are directly involved in GSK3α/β regulation: (1) insulin binds to the insulin receptor and activates the PI3-Akt pathway leading to GSK3α/β inhibition; (2) endoplasmic reticulum stress (ER stress) signaling and/or unfolded protein response (UPR) activation promotes the activation of GSK3α/β through the endoplasmic reticulum kinase (PERK) pathway; and (3) Wnt ligands bind to the Frizzled receptor and induces the formation of a complex of the scaffold protein axin, APC, CK1 and the kinase Dishevelled, which phosphorylates and inactivates GSK3α/β. The complex interplay between these pathways regulates the network of signaling pathways that modulate cell viability and metabolism.

Whole-body GSK3α-deficient mice are viable and develop normally with very mild or no overt phenotype [21]. Genetic deletion of GSK3β results in severe hepatic and cardiac abnormalities during development leading to embryonic lethality [22,23]. Recent evidence suggests that GSK3α and GSK3β play unique and independent roles in skeletal muscle cell insulin signaling [24,25,26], cardiomyocyte development and proliferation [19], and Th cell polarization [26]. GSK3α/β also plays a role in a variety of biological processes, including glycogen metabolism, inflammatory response, migration, proliferation, protein translation, T cell activation, and apoptosis (Figure 2) [17].

GSK3α/β is an atypical kinase as it is usually found in a constitutively active state. It is well established that signaling through the insulin and Wnt pathways inhibit GSK3α/β activity [20,27]. A recent study from our lab shows that the presence of endoplasmic reticulum stress (ER stress) in Thp-1 macrophages activates the protein kinase R-like endoplasmic reticulum kinase (PERK) signaling branch of the unfolded protein response (UPR) to promote GSK3α/β activity [28]. The mechanism underlying this effect is still being delineated. GSK3α/β activity is predominantly regulated by phosphorylation (Figure 2). Autophosphorylation of tyrosine (Tyr) 279 or Tyr216 is required for activation of GSK3α and GSK3β, respectively [17]. Protein kinase B (PKB/Akt) [29], PKA [30], and MAP kinase activated protein (MAPKAP) kinase-1 (p90rsk) [31,32] inhibit GSK3α/β by phosphorylation of serine (Ser) 21 of GSK3α and Ser9 of GSK3β. Protein phosphatase (PP) 1 dephosphorylates Ser21/9 of GSK3α/β and increases GSK3α/β activity [20].

GSK3α/β is also regulated through the formation of distinct protein complexes. GSK3β forms complexes with axin, adenomatous polyposis coli (APC), casein kinase 1 (CK1), and β-catenin, which facilitate the phosphorylation of β-catenin at Thr41, Ser37, and Ser33, leading to its ubiquitylation and degradation [20,27]. Wnt signaling inhibits this complex of proteins, which leads to changes in cellular functions such as cell growth, survival, and differentiation [27,28]. p38MAPK phosphorylates Ser9 of GSK3β and regulates the canonical Wnt–β-catenin signaling pathway [33]. GSK3α/β mainly resides in the cytoplasm but it is also present in mitochondria and the nucleus, as well as other subcellular compartments, where it can be regulated by localized signaling activities [20,27,34,35].

GSK3α/β has been linked to several disorders and diseases, including cancer [36], bipolar mood disorder [37], diabetes [38], Alzheimer’s disease [39], and atherosclerosis [40] (Figure 2). Because of its involvement in a great number of signaling pathways and several disease processes, it is important to better understand the role and regulation of GSK3α/β in different cellular pathways and functions.

**Figure 3:**
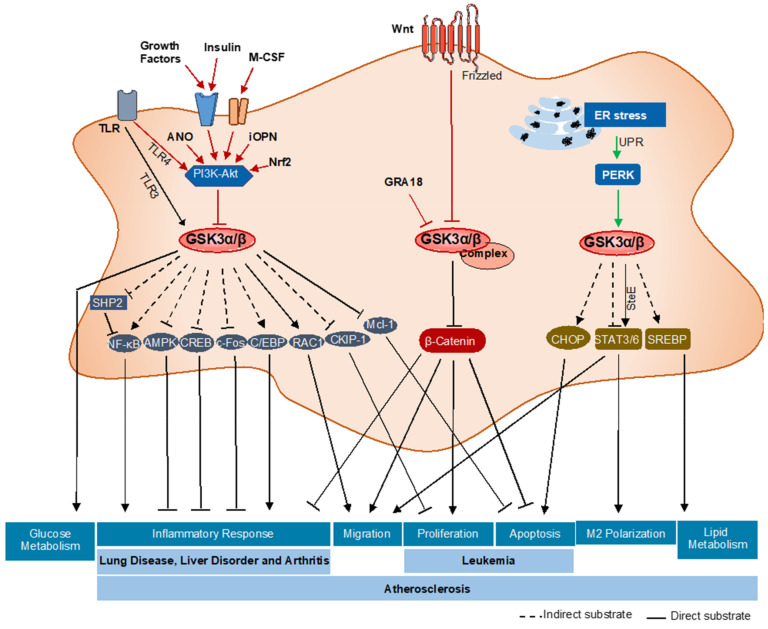
A summary of the GSK3α/β signaling pathways in macrophage functions and related diseases. In macrophages GSK3α/β can be activated or inactivated by different upstream signaling pathways. GSK3α/β has a large number of downstream substrates that regulate a variety of different downstream signaling pathways to control macrophage phenotype and function. Dysregulation of one or more of these pathways has been implicated in the development of several different disorders/diseases.

Evidence suggests that GSK3α/β plays a central role in a variety of different signaling pathways that are relevant to macrophage function including polarization [41,42,43,44], inflammatory response [45,46,47,48,49,50,51,52,53,54,55,56,57], unfolded protein response [28,40,41,42,43], glucose [58,59,60,61] and lipid [28,62] metabolism, viability [17,28,63,64,65], migration [66,67,68], and proliferation [69,70,71,72] (Figure 3). Here, we summarize the current literature on the role of GSK3α/β in regulating specific macrophage functions.

## 3. GSK3α/β—Regulation of Macrophage Function

### 3.1. Macrophage Polarization

In response to various exogenous stimuli, macrophages can polarize into different subtypes, thereby adopting altered functional programs. Polarization pathways are regulated by JAK-STAT signaling (Figure 1). STAT1 is the key transcription regulator for M1 macrophage polarization, whereas STAT3 and STAT6 regulate M2 macrophage polarization [41]. Recent evidence suggests that GSK3α/β modulates macrophage polarization by directly and/or indirectly affecting STAT phosphorylation [42].

Using bone-marrow-derived macrophages (BMDM) from myeloid-specific GSK3α and/or GSK3β knock out mice, we have shown that GSK3α specifically regulates STAT3 and STAT6 phosphorylation/activation. Myeloid-specific GSK3α knockout resulted in increased phosphorylation at Tyr705 of STAT3 in M1 macrophages as well as increased phosphorylation at Tyr641 of STAT6 in M2 macrophages [43]. This suggests that GSK3α actively suppresses STAT3/6 phosphorylation to promote M1 polarization.

The *salmonella* effector (SteE) protein has been shown to alter the substrate and amino acid specificity of GSK3α and β so that they directly phosphorylate Tyr705 of STAT3. This results in STAT3 activation and promotes M2 macrophage polarization [44]. To date, no mammalian equivalent of SteE has been identified.

### 3.2. Inflammatory Response

Macrophages accumulate at sites of injury and participate in the innate immune response, which can be either pro-inflammatory or anti-inflammatory [14]. Several signaling pathways associated with the inflammatory response are known to be regulated by GSK3α/β activity [45,46,47,48]. GSK3α/β plays a role in Toll-like receptor (TLR)-mediated pro-and anti-inflammatory cytokine production. Specifically, inhibition of GSK3α/β results in increased production of anti-inflammatory cytokine (IL-10) and a decrease in pro-inflammatory cytokine (IL-1β, IL-6, TNF-α, and IL-12) production by human peripheral blood mononuclear cells (PBMCs) [45]. A recent review of the role of GSK3β in TLR signaling suggests that GSK3β negatively regulates TLR4-mediated pro-inflammatory cytokine (IFN-β) production [46] and interacts with TRAF3 to act as a positive regulator for TLR3-mediated pro-inflammatory cytokine (IFN-β) production [47]. Cellular growth factors, including insulin, stimulate the class I PI 3-Kinases (PI3K) and activate Akt, which phosphorylates GSK3α/β resulting in its inhibition [48]. Inhibition of GSK3β by PI3K-Akt leads to an increase in IL-10 and IL-12 production [45]. Other data suggest that intracellular osteopontin (iOPN) regulates GSK3β and 4EBP1 phosphorylation via the PI3K-Akt signaling pathway to decrease TLR4-mediated inflammatory responses [49]. Adenosine N1-oxide (ANO) also activates the PI3K-Akt signaling pathway, leading to inhibitory phosphorylation of GSK3β, which results in a decrease in TLR4 mediated pro-inflammatory responses and upregulation of anti-inflammatory transcription factor (c-Fos) [50]. Furthermore, inhibition of GSK3β by the ANO-PI3K–Akt pathway leads to an increase in the binding of cAMP response element-binding protein (CREB) to nuclear coactivator CREB-binding protein (CBP), which results in suppression of the binding of NF-κB p65 to CBP [50]. Together, these data support a role for GSK3β in the TLR4 mediated immune response.

GSK3α/β activity affects several transcription factors that regulate cytokine expression and inflammatory responses [51,52]. GSK3 regulates NF-κB function as GSK3β deficient embryos showed reduced NF-κB function [22]. TNF-α regulates GSK3α/β signaling to promote feedback inhibition of NF-κB, which leads to reduced inflammatory cytokine production [53]. GSK3β inhibits 5′ AMP-activated protein kinase (AMPK) activation and Src homology-2 domain-containing phosphatase (SHP) induction to promote the pro-inflammatory response [54]. Furthermore, this immune regulatory mechanism was independent of PI3K-Akt signaling and GSK3β phosphorylation [55]. GSK3β inhibits SHP2, which indirectly facilitates IFNγ-induced phosphorylation of Ser536 of NF-kB and activation [56]. Another study suggests that GSK3α/β inhibition significantly reduces DNA binding of CCAAT-enhancer-binding proteins (C/EBP) and increases IL-10/IL-12 production in granulocyte-macrophage/dendritic cells (GM/DCs) [57]. The parasite-dense granule protein GRA18 forms a complex with GSK3α/β and PP2A-B56 in the cytoplasm, which drives β-catenin accumulation and increases chemokine (C-C motif) ligand (CCL) 17 and CCL22 chemokine production, leading to an increase in the anti-inflammatory response [57]. In summary, GSK3α/β, directly and indirectly, regulates the inflammatory response in macrophages. The downstream substrate(s) that link GSK3α/β and these factors require further investigation to better understand the relevant signaling pathways.

### 3.3. Unfolded Protein Response

In response to ER stress, macrophages activate the UPR to maintain ER homeostasis. Evidence from our lab and others has shown that ER stress can induce GSK3α/β activity [28,40]. ER stress signaling through GSK3α/β is dependent upon the PERK branch of the UPR. ER stress-GSK3α/β signaling appears to regulate downstream pathways involving apoptosis/viability, polarization, and lipid accumulation [28,43]. The mechanisms by which PERK promotes GSK3α/β are still unknown and require further investigation.

### 3.4. Glucose Metabolism

GSK3α/β was named for its role in regulating glycogen synthesis/metabolism. Whereas anti-inflammatory macrophages rely predominantly on oxidative phosphorylation for energy production, pro-inflammatory macrophages rely on the glycolytic pathway. The regulation of glucose metabolism in the inflammatory response is not fully understood. The nuclear factor erythroid 2-related factor 2 (Nrf2) is a transcription factor that coordinates glucose metabolism to stress responses, and specifically upregulates antioxidant response elements in conditions of oxidative stress [58,59,60]. Genetic Nrf2 knockout in M1 macrophages downregulates the expression of Akt and thereby reduces the inhibitory phosphorylation of GSK3β. Active GSK3β directly promotes the inhibition of glycogen synthase and reduces glycogenesis [61].

### 3.5. Lipid Accumulation/Metabolism

Lipid metabolism plays a critical role in the function of both pro-and anti-inflammatory macrophages [62]. In particular, anti-inflammatory macrophages display enhanced mitochondrial oxidative phosphorylation (OXPHOS) [62]. During atherogenesis, macrophages can accumulate lipids and become foam cells. A previous study from our lab suggests that pharmacological inhibition of GSK3α/β attenuates the expression of genes regulating lipid and cholesterol biosynthesis, including fatty acid synthase (FAS), sterol regulatory element-binding proteins (SREBP)-1c, SREBP-2, and 3-hydroxy-3-methylglutaryl-CoA (HMG-CoA) [28]. Furthermore, inhibition of GSK3α/β in vivo blocks the ability of macrophage foam cells to accumulation lipid and attenuates atherogenesis [28]. The role of GSK3α/β in reverse cholesterol transport is still unknown and requires further investigation.

### 3.6. Apoptosis

GSK3α/β has previously been shown to play a role in regulating cell viability [17]. Data from our lab and others suggest that GSK3α/β plays a pro-apoptotic role in cells. GSK3α/β inhibition decreases the expression of the pro-apoptotic C/EBP homologous protein (CHOP) in macrophages [28]. Activated GSK3α/β phosphorylates myeloid cell leukemia (Mcl)-1, resulting in Mcl-1 degradation followed by apoptosis [63]. In U937 cells (an acute myeloid leukemia cell line), a complex of N-Myc downstream-regulated gene 2 (NDRG2), GSK3α/β, and PP2A is formed upon treatment with the anti-cancer drug As2O3 [64]. This leads to GSK3α/β activation through dephosphorylation at Ser9 by PP2A, followed by Mcl-1 degradation and apoptosis [65]. In alveolar macrophages, LPS-induced apoptosis occurs by activation of the Wnt pathway followed by destabilization of GSK3β and accumulation of phospho-Ser9-GSK3β and β-catenin [65]. These data suggest that GSK3α/β indirectly activates CHOP and directly phosphorylates β-catenin and Mcl-1 to allow macrophages to undergo apoptosis.

### 3.7. Migration

The ability of macrophages to move is central to their role in the innate immune system. Macrophage movement is essential for the phagocytosis of foreign material and apoptotic cells. Studies have shown that in obese visceral adipose tissue (VAT), GSK3 inhibition reverses obesity-induced inflammation via reducing apoptosis inhibitor of macrophage (AIM) levels to attenuate macrophage/monocyte migration and macrophage accumulation [66]. In LPS stimulated macrophages, Akt inhibits GSK3β, which leads to β-catenin accumulation. This activates matrix metalloproteinase (MMP)-9 gene induction and promotes cell migration [67]. Inhibition of GSK3β affects integrin signaling via reduced Ras-related C3 botulinum toxin substrate (RAC)-1 activity, thereby affecting the activation of cofilin and actin rearrangement. These activities lead to a decrease in lamellipodia formation, adhesion, and migration of monocytes, thereby preventing monocyte migration across brain endothelial cells [68]. In summary, GSK3α/β signals through STAT, β-catenin, and RAC1 to regulate macrophage migration.

### 3.8. Proliferation

In response to inflammation, macrophages accumulate and proliferate at the injury location in the tissue. These macrophages may be recruited from the blood or be derived from resident macrophages. Macrophage colony-stimulating factor (M-CSF) signals through PI3K-Akt to inhibit GSK3β, resulting in casein kinase 2 interacting protein-1 (CKIP-1) and β-catenin accumulation in the cytosol [69]. β-catenin promotes the expression of proliferation genes such as cyclin D and c-Myc. CKIP-1 inhibits TNF receptor associated factor (TRAF) 6-mediated Akt activation, acting as a negative feedback loop [69]. PI3K/Akt/GSK3 signaling has been shown to play a central role in the proliferation of Anthrax lethal toxin (LeTx)-induced macrophages [70]. GSK3β inhibitors suppress cell growth and induce apoptosis in different leukemia cell lines including acute myeloid leukemia (AML) [71]. Ceramide 1-phosphate (C1P) upregulates the expression of two major downstream targets of GSK3β, cyclin D1 and c-Myc, which regulate cell proliferation [72]. C1P triggered rapid phosphorylation of PI3K-Akt, which induces GSK3β inhibition, leads to an increase in macrophage proliferation [72]. These data indicate that GSK3α/β phosphorylates β-catenin and regulates proliferation-related genes such as cyclin D and c-Myc to control macrophage proliferation.

In summary, GSK3α/β activity is positively regulated by the upstream ER stress-PERK pathway. Other upstream signaling factors, including ANO, GRA18, Wnt signaling pathway, and the insulin-PI3K-Akt pathway, led to GSK3α/β inactivation in macrophages. GSK3α/β signals through downstream effector proteins, including Glut4, STAT, NF-κB, CREB, C/EBP, Mcl-1, β-catenin, CHOP, Mcl-1, SREBP, and RAC1, to regulate a variety of functions in macrophages.

## 4. Diseases Associated with Macrophage Dysfunction and GSK3α/β

GSK3α/β activity has been implicated in the pathogenesis of several different metabolic diseases and disorders. Therefore, it is important to understand the specific roles of GSK3α/β and the therapeutic potential of specific interventions to target this factor (Figure 3).

In vivo studies show that inhibition of GSK3β decreases production of TNF-α and macrophage inflammatory protein (MIP)-2 as well as the release of the alarmins high mobility group box (HMGB)-1 and histone 3 in the lungs, perhaps reducing the severity of LPS-induced lung injury [73]. These results show that GSK3β plays an important contributory role in worsening the severity of acute lung injury (ALI). In alveolar macrophages from pulmonary fibrosis patients and mice, GSK3β and the ubiquitin-editing enzyme A20 regulate C/EBPβ enzymatic activity and play a role in lung fibrosis [74]. This study suggests that GSK3β is a potential target for treating pulmonary fibrosis and fibroproliferative lung diseases.

In bone marrow-derived macrophages, inhibition of GSK3β down-regulates pro-inflammatory gene expression including IL12, TNF-α, and C-X-C motif chemokine (CXCL) 10, which protect the liver against ischemia/reperfusion injury (IRI) [75]. These data suggest that GSK3β may be a target as a therapeutic strategy to ameliorate liver IRI.

A previous study suggests that GSK3α/β inhibitors could be used as anti-inflammatory drugs to treat the rheumatoid arthritis (RA) [76]. Another study in a mouse model of rheumatoid arthritis shows that GSK-3β inhibitors suppress inflammatory responses by downregulating the NF-kB signaling pathway, along with downregulating the expression of c-Jun N-terminal kinase (JNK), c-jun, activating transcription factor (ATF) 2, and p-38 [77]. These findings suggest that the GSK3β may be an efficient therapeutic target for RA.

Recent studies from our lab have implicated GSK3α in the progression and development of atherosclerosis [43]. Specifically, genetic ablation of myeloid GSK3α attenuates the progression of atherosclerosis in low-density lipoprotein receptor (Ldlr) knockout mice [43]. These studies suggest that the specific inhibition of one isoform of GSK3 may be an effective therapeutic approach to treat atherosclerosis. Small molecule GSK3 isoform-specific inhibitors have recently been identified and tested in mouse model systems [78]. GSK3α-specific inhibition weakens leukemia initiation and prolongs survival in acute myeloid leukemia (AML) mouse models [78]. This study suggests the possibility of using small molecules targeting GSK3α as a therapeutic tool in the treatment of atherosclerosis and AML.

In summary, the central regulatory role of GSK3α/β in macrophage viability and immunometabolic function suggests that it may be a viable target to treat a variety of diseases and disorders related to cancer and inflammation. Currently, the therapeutic targeting of GSK3 is impeded by several factors. First, it is clear that GSK3α/β plays a central role in many important pathways and that inhibition of these factors could have serious detrimental side effects. Second, we have a limited understanding of the specific roles of GSK3α and GSK3β in health and disease. Third, until recently there were no small molecule inhibitors that could distinguish between GSK3α and GSK3β. The identification of specific GSK3α and GSK3β inhibitors [78] may allow for the more precise targeting of the relevant isoform in the treatment of a specific disease or disorder in a way that limits unwanted detrimental side effects. The next few years will provide many exciting answers as these and other more specific interventions are tested.

## 5. Translational Benefits

An understanding of the role of GSK3α/β-signaling in macrophages is potentially important in the field of drug discovery and the treatment of disease. Currently, there are 31 clinical studies on GSK3α/β taking place all over the world [79]. These are primarily focused upon the treatment of conditions and diseases ranging from cancer to neurological disorders. None of the ongoing clinical trials are specifically focused upon macrophage function or associated disease. Results from preclinical studies suggest that isoform-targeted inhibition of macrophage GSK3α or GSK3β may be effective in the treatment of acute myeloid leukemia [78] and atherosclerosis [40,43]. Isoform-specific inhibitors will be important in future clinical trials examining efficacy in the treatment of diseases including cancer and cardiovascular diseases.

## 6. Conclusions

In this review, we have summarized the results from recent reports that address how GSK3α/β is regulated in macrophages and how GSK3α/β modulates different macrophage functions and related diseases. Evidence suggests that GSK3α/β directly or indirectly affects different downstream molecules to regulate virtually all macrophage functions. More investigation is needed to fully understand all signaling pathways related to GSK3α/β and macrophages functions. This knowledge will potentially facilitate the development and testing of new therapeutics to treat a variety of immunometabolic diseases.

## Data Availability

No new data were created or analyzed in this study. Data sharing is not applicable to this article.

## Abbreviations

AIM	Apoptosis inhibitor of macrophage
ALI	Acute lung injury
AML	Acute myeloid leukemia
AMPK	5′ AMP-activated protein kinase
ANO	Adenosine N1-oxide
AP1	Activator protein 1
APC	Adenomatous polyposis coli
ATF	Activating transcription factor
BMDM	Bone marrow derived macrophages
C/EBP	CCAAT-enhancer-binding proteins
C1P	Ceramide 1-phosphate
CBP	CREB-binding protein
CCL	Chemokine (C-C motif) ligand
CHOP	C/EBP Homologous Protein
CK1	Casein kinase 1
CKIP-1	Casein kinase 2 interacting protein-1
CREB	Cyclic AMP response element binding protein
CXCL	C-X-C motif chemokine
ER Stress	Endoplasmic reticulum stress
FAS	Fatty acid synthase
GSK3	Glycogen synthase kinase 3
HMGB	High mobility group box
HMGCoA	3-hydroxy-3-methylglutaryl-CoA
IFN	Interferon
IFNR	Interferon receptor
IL	Interleukin
IL4R	Interleukin 4 receptor
iOPN	Intracellular osteopontin
IR	Insulin receptor
IRI	Ischemia/reperfusion
IRS	Insulin receptor substrate
JNK	c-Jun N-terminal kinase
Ldlr	Low-density lipoprotein receptor
LeTx	Lethal toxin
LPS	Lipopolysaccharides
LRP	Lipoprotein receptor—related protein
LXR	Liver X receptors
MAPKAP	MAP kinase activated protein
Mcl-1	Myeloid cell leukemia 1
MIP	Macrophage inflammatory protein
MMP	Matrix metalloproteinase
NDRG2	N-Myc downstream-regulated gene 2
NFκB	Nuclear factor kappa-light-chain-enhancer of activated B cells
NR	Nuclear receptor
PBMC	Peripheral blood mononuclear cells
PERK	Protein kinase R-like endoplasmic reticulum kinase
PI3K	Phosphoinositide 3-kinases
PKB	Protein kinase B
PP	Protein phosphatase
PPARγ	Peroxisome proliferator-activated receptor gamma
RA	Rheumatoid arthritis
RAC1	Ras related C3 botulinum toxin substrate 1
Ser	Serine
SHP	Src homology-2 domain-containing phosphatase
SREBP	Sterol regulatory element-binding proteins
STAT	Signal transducer and activator of transcription
SteE	*Salmonella* effector
TGFβ	Transforming growth factor
TLR	Toll-like receptor
TNFɑ	Tumor necrosis factor ɑ
TRAF	TNF receptor associated factor
Tyr	Tyrosine
VAT	Visceral adipose tissue
